# Neuroprotective Effects of Collagen-Glycosaminoglycan Matrix Implantation following Surgical Brain Injury

**DOI:** 10.1155/2019/6848943

**Published:** 2019-01-27

**Authors:** Jia-Hui Chen, Wei-Cherng Hsu, Kuo-Feng Huang, Chih-Huang Hung

**Affiliations:** ^1^Institute of Medical Sciences, Tzu Chi University, Hualien, Taiwan; ^2^Division of General Surgery, Department of Surgery, Taipei Tzu Chi Hospital, Buddhist Tzu Chi Medical Foundation, New Taipei City, Taiwan; ^3^School of Medicine, Buddhist Tzu Chi University, Hualien, Taiwan; ^4^Department of Ophthalmology, Taipei Tzu Chi Hospital, Buddhist Tzu Chi Medical Foundation, New Taipei City, Taiwan; ^5^Division of Neurosurgery, Department of Surgery, Taipei Tzu Chi Hospital, Buddhist Tzu Chi Medical Foundation, New Taipei City, Taiwan; ^6^Department of Research, Buddhist Tzu Chi General Hospital, Hualien, Taiwan

## Abstract

**Background:**

Neurological deficits following neurosurgical procedures are inevitable; however, there are still no effective clinical treatments. Earlier reports revealed that collagen-glycosaminoglycan (CG) matrix implantation promotes angiogenesis, neurogenesis, and functional recovery following surgical brain injury (SBI). The present study was conducted to further examine the potential neuroprotective effects of collagen-glycosaminoglycan (CG) matrix implantation following neurosurgery.

**Methods:**

CG implantation was performed in the lesion cavity created by surgical trauma. The Sprague-Dawley rat model of SBI was used as established in the previous study by the author. The rats were divided into three groups as follows: (1) sham (SHAM), (2) surgery-induced lesion cavity (L), and (3) CG matrix implantation following surgery-induced lesion cavity (L+CG). Proinflammatory (tumor necrosis factor-alpha (TNF-*α*), interleukin-6 (IL-6), and NF-*κ*B (nuclear factor kappa-light-chain-enhancer of activated B cells)) and anti-inflammatory (IL-10 and granulocyte-macrophage colony-stimulating factor (GMCSF)) cytokine expression was evaluated by enzyme-linked immunosorbent assays. Microglial activation was evaluated by immunohistochemistry, and the neuroprotective effect of CG matrix implantation was evaluated by an immunohistochemical study of microglia ED-1 and IBA-1 (activated microglia) and myeloperoxidase (MPO) and by the analysis of IL-6, IL-10, TNF-*α*, NF-*κ*B, and GMCSF cytokine levels. Apoptosis was also assessed using a TUNEL assay.

**Results:**

The results showed that CG matrix implantation following surgically induced lesions significantly decreased the density of ED-1, IBA-1, and MPO (activated microglia). The tissue concentration of proinflammatory cytokines, such as TNF-*α*, IL-6, and NF-*κ*B was significantly decreased. Conversely, the anti-inflammatory cytokines GMCSF and IL-10 were significantly increased.

**Conclusions:**

Implantation of the CG matrix following SBI has neuroprotective effects, including the suppression of microglial activation and the production of inflammatory-related cytokines.

## 1. Introduction

Surgical brain injury (SBI) can cause disability after brain surgery. The most common clinical symptom following SBI is long-term neurobehavioral deficits. However, a few pharmaceutical treatments can improve these neurobehavioral defects. Treatment strategies include increased nerve regeneration and reduced inflammation during central nervous system (CNS) recovery. However, the therapeutic strategies for clinical SBI are limited due to the irreversibility of injuries to neural structures [1]. Several recent studies have used new biological matrix materials to repair nerve damage. The CG matrix is a type of semisynthetic collagen matrix made from bovine deep flexor tendon, which has delicate mechanical properties. Collagen matrices, such as the CG matrix, have been commonly used for other types of tissue. The previous reports have proposed that the CG matrix serves as a moderator of cell behaviors, such as adhesion, growth, and differentiation, as well as offering structural support [2–4].

Several recent studies have explored the use of collagen matrices and biological scaffold extracellular matrices (ECMs) in the repair of neural injuries following surgery [3, 4]. In the past, biological matrix materials that release growth factors have been used to improve nerve regeneration. The recent studies have shown that the implantation of CG matrices may lead to the promotion of angiogenesis, neurogenesis, and functional recovery after SBI [3, 4]. The current study is aimed at determining whether the implantation of biological matrix materials can have other beneficial effects at the site of SBI in rats. The results revealed that collagen matrices may reduce the extent of neurological damage and provide neuroprotective effects.

## 2. Materials and Methods

### 2.1. Animals

All of the procedures and experimental protocols involving animals in the present study were approved by the Animal Care and Use Committee of Taipei Tzu Chi Hospital, Buddhist Tzu Chi Medical Foundation (approval no. 107-IACUC-002), and all protocols were performed in accordance with the guidelines set by the National Institutes of Health Guide for the Care and Use of Laboratory Animals.

### 2.2. Animal Model of SBI and Experimental Design

Adult male Sprague-Dawley rats weighing 300-350 g were anesthetized by intraperitoneal injection of pentobarbital (65 mg/kg) and placed in the prone position in a stereotaxic apparatus. Surgery was performed under sterile conditions. The animals were then placed into one of the following groups: (i) The sham group (SHAM), animals received only a craniotomy and replacement of the bone flap. (ii) The lesion group (L), after a craniotomy and exposure of a square window displaying the underlying right frontal lobe of the brain covered by the dura, the dura was carefully incised with a no. 20 needle to minimize bleeding, and a frontal-parietal lesion (6 × 4 mm edge) was localized from 1.0 mm anterior to 4.0 mm posterior of the bregma and 1.0 to 5.0 mm lateral to the midline. The depth of the lesion was 3.0 mm from the brain surface. (iii) The lesion with CG group (L+CG), after undergoing the same procedure as the L group, a block of CG matrix was placed into the lesion cavity, which was formed by tissue removal from the frontal-parietal area. There were ten rats in each group (*n* = 10). All procedures used sterile gauze pads and saline irrigation to control bleeding, and the skin was sutured using 3-0 silk (Ethicon, Taipei, Taiwan).

### 2.3. CG Preparation and Assessment

A biodegradable collagen matrix of a 1% collagen and 0.02% glycosaminoglycan copolymer was produced as previously described with several modifications [5–7]. In brief, a coprecipitate of type I porcine tendon (SPF, Animal Technology Institute, Taiwan) and chondroitin 6-sulfate (Sigma Chemical Company, St. Louis, MO) in 0.05 M acetic acid was freeze-dried to yield a highly porous sheet 4 mm in thickness. The freeze-drying process yielded a network of the CG copolymer with approximately 95% pore volume fraction and average pore diameter of 140 ± 20 mm. The sheets of the copolymer were cross-linked by dehydrothermal treatment consisting of exposure to a vacuum at a temperature of 105°C for 24 h, followed by exposure to UV light [8]. Disks of the CG copolymer, 6 × 4 × 3 mm (72 mm^3^) in size, were cut by trephination and further cross-linked by immersion in 0.25% aqueous glutaraldehyde for 24 h. Residual glutaraldehyde was removed by exhaustive rinsing in multiple changes of phosphate-buffered saline (PBS) over 48 h. There is no hyaluronic acid within the scaffold. The matrix was designated with an optimal degradation time of ~28 days, in line with the time course of endogenous neural stem cell proliferation and differentiation [9].

### 2.4. Immunohistochemistry

The rats were anesthetized as above and transcardially perfused with phosphate-buffered saline (PBS), followed by 4% paraformaldehyde on day (D) 7, D14, D21, and D28. The brains were removed and fixed in 4% paraformaldehyde overnight, then embedded in paraffin blocks. The slices were collected at +1.0, 0.0, and -1.0 mm (from the center of the hemorrhagic lesion) anterior and posterior to the bregma using a cryostat (Leica CM 1900). Serial sections (6 *μ*m) through the cerebral cortex were processed for double immunohistochemistry staining.

Paraffin-embedded sections were mounted on slides, deparaffinized, and rehydrated. Immunohistochemical analysis was performed after heat-mediated antigen retrieval. Hydrogen peroxide (3% in distilled water) was used to block endogenous peroxidase activity. The sections were blocked in PBS containing 1% goat serum and 0.3% Triton X-100 for 1 hour, followed by incubation with primary antibodies against ED-1 (1 : 100; Thermo Fisher Scientific, Waltham, MA, USA) and myeloperoxidase (MPO; 1 : 100; Abcam, Cambridge, UK) for 2 h at room temperature and followed by poly-horseradish peroxidase (HRP) anti-mouse secondary antibodies for 30 min. For the negative control, primary antibodies were omitted. Peroxidase activity was visualized using 0.5 mg/mL 3,3′-diaminobenzidine (brown color) with 0.05% H_2_O_2_ as the activator. The sections were counterstained with hematoxylin and examined by light microscopy. The positive cells were counted in six squares (1 mm^2^) randomly located around the lesion on each slice. In the SHAM group, we randomly defined 6-square (1 mm^2^) area in each 9 slices, where density (cells/mm^2^) of ED-1^+^ and MPO^+^ cells was counted. In the L group, we randomly defined 6-square (1 mm^2^) area along lesion boundary zone (LBZ) near cavity in each 9 slices, where density (cells/mm^2^) of ED-1^+^ and MPO^+^ cells was counted. In the L+CG group, we randomly defined 6-square (1 mm^2^) area along LBZ near intramatrix zone (IMZ) in each 9 slices, where density (cells/mm^2^) of ED-1^+^ and MPO^+^ cells was counted. An average cell number in each plane (total 6 mm^2^) was calculated from 54 squares in 9 planes. An independent investigator performed all cell counting.

### 2.5. Immunofluorescence Staining

Each section was treated with 4% paraformaldehyde in PBS for 1 h at room temperature, then washed twice with PBS and incubated in 2 M HCl at 37°C for 1 h for immunofluorescence staining. The sections were subsequently incubated in blocking solution with mouse monoclonal anti-GFAP (1 : 1,000; EMD Millipore, Billerica, MA, USA) or rabbit polyclonal anti-IBA-1 primary antibodies (1 : 50; Abcam, Cambridge, UK) overnight at 4°C. The sections were then incubated with Alexa Fluor-488 goat anti-mouse immunoglobulin G (IgG; 1 : 200; Invitrogen; Thermo Fisher Scientific Inc.) and DyLight 549 anti-rabbit IgG (1 : 200; Jackson ImmunoResearch Laboratories Inc., West Grove, PA, USA) secondary antibodies at room temperature for 2 h. The sections were counterstained with DAPI and mounted with Mounting Medium H-1000 (Vector Laboratories Inc., Burlingame, CA, USA). Negative control was established by omitting the primary antibodies. Fluorescent microscopy images were obtained using a Nikon ellipse 80i microscope (Nikon Corporation, Tokyo, Japan) and a Nikon Digital Sight DS-5M camera, using the NIS-Elements F 2.30 software (Nikon Corporation). Digital image processing was performed by Image-Pro Plus, version 5.1.

### 2.6. Cell Counting

Following double immunofluorescence staining, the positively stained cells in the intramatrix zone (IMZ) and the lesion boundary zone (LBZ) were counted manually on D7, D14, D21, and D28 in three to five different fields per section of each rat brain (using an eyepiece grid covering an area of 0.0625 mm^2^). An individual who was blinded to the experimental design performed the counting. Vessels and blood cells were excluded. Sections were observed, and images were acquired using a Nikon epifluorescent microscope. The total cells in the LBZ were visualized by DAPI staining at 20x magnification using the Openlab software (Improvision, Cambridge, MA, USA). IBA-1 cells were visualized as fluorescent red (DL 549) and GFAP cells were visualized as fluorescent green (Alexa 488) in the LBZ at 20x magnification. The double-positive cells were visualized as yellow. For double immunofluorescence staining, the double-positive cells were also manually counted in the same way. The results were presented as the number of immunopositive cells per field.

#### 2.6.1. Terminal Deoxynucleotidyl Transferase Nick-End Labeling (TUNEL) Assay

The apoptotic cells in brain tissue samples were detected using an In Situ Cell Death Detection kit (Roche, Switzerland) according to the manufacturer's instructions. Briefly, paraffin-embedded specimens were cut into 4–5 *μ*m thickness sections. After deparaffinization and washing in PBS, the sections were treated with proteinase K, then incubated with TUNEL reaction mixture at 37°C for 1 h. Slides were washed with PBS and then treated with HRP and DAB terminated until the color was developed. Apoptotic index was determined by counting the number of TUNEL-positive cells. Eight slides per block were evaluated. For each slide, 8 fields were randomly chosen and 100 cells per field were counted and calculated for the apoptosis index.

#### 2.6.2. Enzyme-Linked Immunosorbent Assay (ELISA) Analysis of GMSF, TNF-*α*, IL-6, IL-10, and NF-*κ*B in the Brain Lesion Site Tissues

Brains from rats in the SHAM, L, and L+CG groups were removed after cervical dislocation on D7, D14, D21, and D28 after surgery. A 3 mm coronal section was taken from the injured area over the parietal cortex, snap-frozen in liquid nitrogen, and stored at -70°C until use. All brain samples were homogenized in a buffer consisting of 0.05 M Tris HCl, 0.15 M NaCl, 0.1% Nonidet 40, 0.5 M phenylmethylsulfonyl fluoride, 50 mg/mL aprotinin, 10 mg/mL leupeptin, 50 mg/mL pepstatin, 4 mM sodium orthovanadate, 10 mM sodium fluoride, and 10 mM sodium pyrophosphate. Homogenates were centrifuged at 12,000 ×g for 15 min at 4°C. The supernatants were then removed and assayed in duplicate using TNF-*α*, IL-6, and IL-10 assay kits (R&D Systems Inc., Minneapolis, MN, USA), NF-*κ*B assay kits (Imgenex, San Diego, CA, USA), and a granulocyte-macrophage colony-stimulating factor (GMCSF) assay kit (MyBioSource, CA, USA) according to the manufacturer's protocol. Concentrations of the tissue proteins (GMCSF, TNF-*α*, IL-6, and IL-10) were expressed as picograms of antigen per milligram of protein, and NF-*κ*B was expressed as nanograms of antigen per milligram of protein. The concentration of GMCSF, TNF-*α*, IL-6, IL-10, and NF-*κ*B in brain lesion site tissue samples was measured on D7, D14, D21, and D28 using ELISA kits.

### 2.7. Statistical Analysis

Data were analyzed using the SPSS 24.0 statistical software and expressed as the mean ± standard deviation (SD). Student's *t*-test was performed for the comparison of two groups. Statistical comparisons between multiple groups were performed using one-way analysis of variance (ANOVA), and multiple time points were analyzed by two-way ANOVA with Bonferroni's correction. In all cases, *n* refers to the number of animals in the group. The significance level was set at 0.05, and *p* < 0.05 was considered to indicate a statistically significant difference.

## 3. Results

### 3.1. ED-1^+^ Cells Decreased following Implantation of the CG Matrix

**Figure 1:**
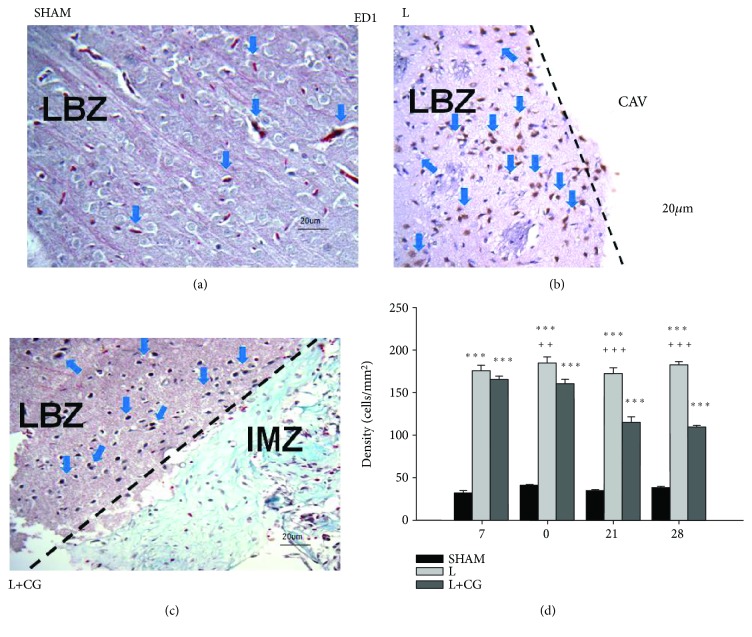
Evidence of a sustained decrease in inflammatory ED1^+^ cells (blue arrows) in the lesion boundary zone (LBZ) region of surgical brain lesions in the collagen-glycosaminoglycan matrix implantation following surgery-induced lesion cavity (L+CG) group compared with the surgery-induced lesion cavity (L) group. Representative microphotographs of immunofluorescence staining in representative brain sections from (a) the sham (SHAM) group, (b) the L group, and (c) the L+CG group on day 21. (d) Number of ED1^+^ cells in the LBZ from brain sections from the SHAM, L, and L+CG groups on days 7, 14, 21, and 28 after surgery. Data are presented as the mean ± SD (standard deviation). ^∗^*p* < 0.05, ^∗∗^*p* < 0.01, and ^∗∗∗^*p* < 0.001 vs. the SHAM group; ^+^*p* < 0.05, ^++^*p* < 0.01, and ^+++^*p* < 0.001 vs. the L+CG group. LBZ: lesion boundary zone; CAV: cavity; IMZ: intramatrix zone.

Representative photomicrographs show double immunofluorescence staining with antibodies against ED-1, in brain sections from the SHAM, L, and L+CG groups on D21 following injury (Figure 1). The density (cells/mm^2^) of ED-1^+^ cells was counted in the LBZ of the SHAM, L, and L+CG groups of rats at various time points. A significant decrease in ED-1^+^ cells was observed in the L+CG group against the L group on D7 after the surgically induced brain lesion; the levels remained stable with a slight increase on D28.

### 3.2. IBA-1^+^ Cells Decreased following Implantation of the CG Matrix

**Figure 2:**
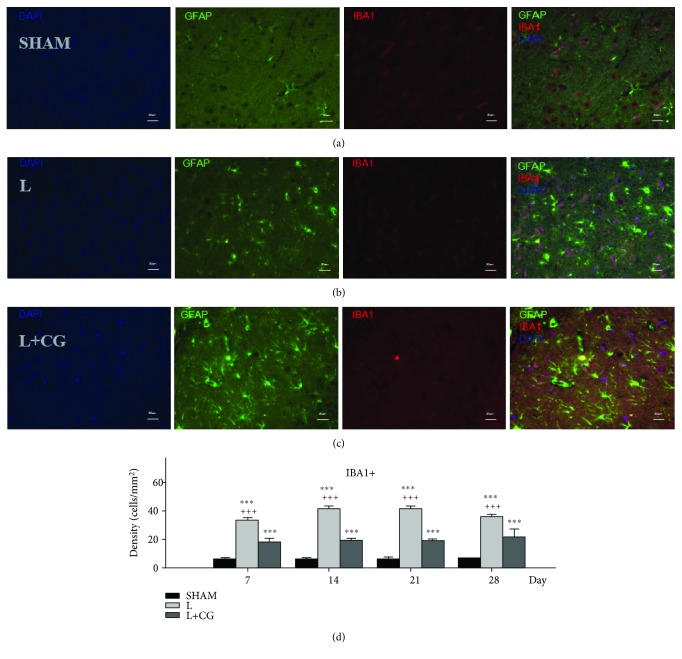
Identification of a sustained decrease in IBA-1^+^ cells in the lesion boundary zone (LBZ) of surgical brain lesions following implantation of the collagen-glycosaminoglycan (CG) matrix. Representative microphotographs of double immunofluorescence staining of representative brain sections from the (a) sham (SHAM) group, (b) the surgery-induced lesion cavity (L) group, and (c) the CG matrix implantation following surgery-induced lesion cavity (L+CG) group on day 14 following surgical brain trauma. In the merged image of immunoreactivity, GFAP (green) represents the astrocyte marker and IBA-1 (red) indicates positive activity in cells. (d) Numbers of IBA-1^+^ cells in LBZ from the brain sections in the SHAM, L, and L+CG groups on days 7, 14, 21, and 28 after surgery. Data are presented as the mean ± standard deviation. ^∗^*p* < 0.05, ^∗∗^*p* < 0.01, and ^∗∗∗^*p* < 0.001 vs. the SHAM group; ^+^*p* < 0.05, ^++^*p* < 0.01, and ^+++^*p* < 0.001 vs. the L+CG group.

IBA-1 is commonly used as a marker of activated microglia [10]. Therefore, to determine whether the activation of microglia cells was correlated with neuroinflammation, the density of IBA-1^+^ cells within the LBZ was examined (Figure 2). The density (cells/mm^2^) of IBA-1^+^ cells in the LBZ of the SHAM, L, and L+CG groups was also measured at various time points. The L+CG group showed a significant decrease in IBA-1^+^ cells against the L group on D7 following the surgical brain lesion; the levels remained stable with a slight decrease on D28.

### 3.3. MPO^+^ Cells Decreased following Implantation of the CG Matrix

**Figure 3:**
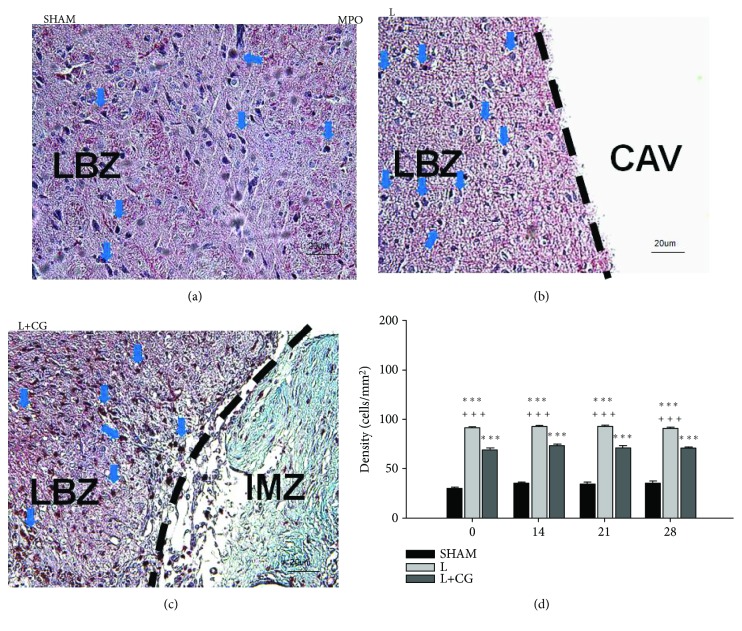
Sustained significant decrease in MPO^+^ cells (blue arrows) in the lesion boundary zone (LBZ) of surgical brain lesions in the collagen-glycosaminoglycan matrix implantation following surgery-induced lesion cavity (L+CG) group compared with the surgery-induced brain lesion (L) group. Representative microphotographs of immunofluorescence staining in representative brain sections from (a) the sham (SHAM) group, (b) the L group, and (c) the L+CG group on day 21. (d) Number of MPO^+^ cells in the LBZ in brain sections from the SHAM, L, and L+CG groups on days 7, 14, 21, and 28 after surgery. Data are presented as the mean ± standard deviation. ^∗^*p* < 0.05, ^∗∗^*p* < 0.01, and ^∗∗∗^*p* < 0.001 vs. the SHAM group; ^+^*p* < 0.05, ^++^*p* < 0.01, and ^+++^*p* < 0.001 vs. the L+CG group. LBZ: lesion boundary zone; CAV: cavity; IMZ: intramatrix zone.

To evaluate the activity of MPO, the presence of MPO^+^ cells was determined in the LBZ of surgically induced brain lesions following implantation of the CG scaffold. The L+CG group showed significant decreases in MPO^+^ cells in the LBZ against the L group on D7, which continued to decrease up to D28 (Figure 3).

### 3.4. TUNEL^+^ Cells Decreased in the LBZ following Implantation of the CG Matrix

**Figure 4:**
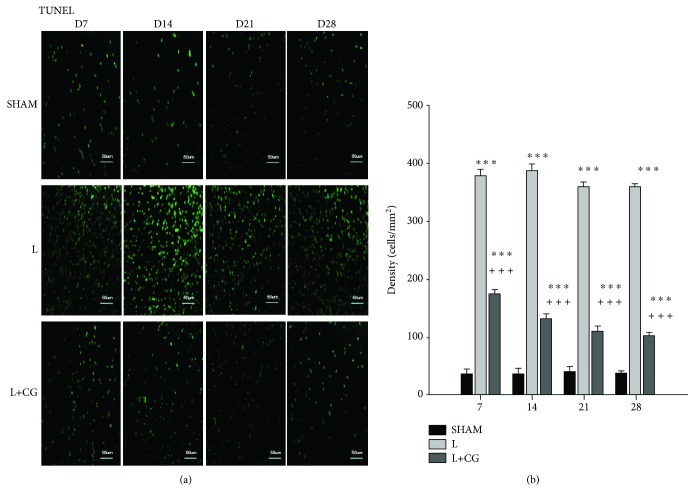
Early decrease of TUNEL^+^ cells in the lesion boundary zone (LBZ) of surgical brain lesions following implantation of the collagen-glycosaminoglycan (CG) matrix. (a) Representative microphotographs of double immunofluorescence staining of representative brain sections from the sham (SHAM), surgery-induced brain lesion (L), and CG matrix implantation following surgery-induced lesion cavity (L+CG) groups on day 14 following surgical brain trauma. In the image of immunoreactivity, TUNEL (green) was used to quantify the apoptotic cells in the SHAM, L, and L+CG groups. (b) Number of TUNEL^+^ cells in the LBZ in brain sections from the SHAM, L, and L+CG groups on days 7, 14, 21, and 28 after surgery. Data are presented as the mean ± standard deviation. ^∗^*p* < 0.05, ^∗∗^*p* < 0.01, and ^∗∗∗^*p* < 0.001 vs. the SHAM group; ^+^*p* < 0.05, ^++^*p* < 0.01, and ^+++^*p* < 0.001 vs. the L+CG group.

Double immunofluorescent staining of TUNEL^+^ cells was performed to evaluate cell damage and identify cells in the last phase of apoptosis. The density (cells/mm^2^) of TUNEL^+^ cells in the LBZ of the SHAM, L, and L+CGM groups of rats was measured at different time points. The L+CG group showed a significant decrease in TUNEL^+^ cells on D7, D14, D21, and D28 following surgical induction of a brain lesion compared with the L group (Figure 4).

### 3.5. TNF-*α*, IL-6, and NF-*κ*B Levels Decreased and GMCSF and IL-10 Levels Increased following Implantation of the CG Matrix

**Figure 5:**
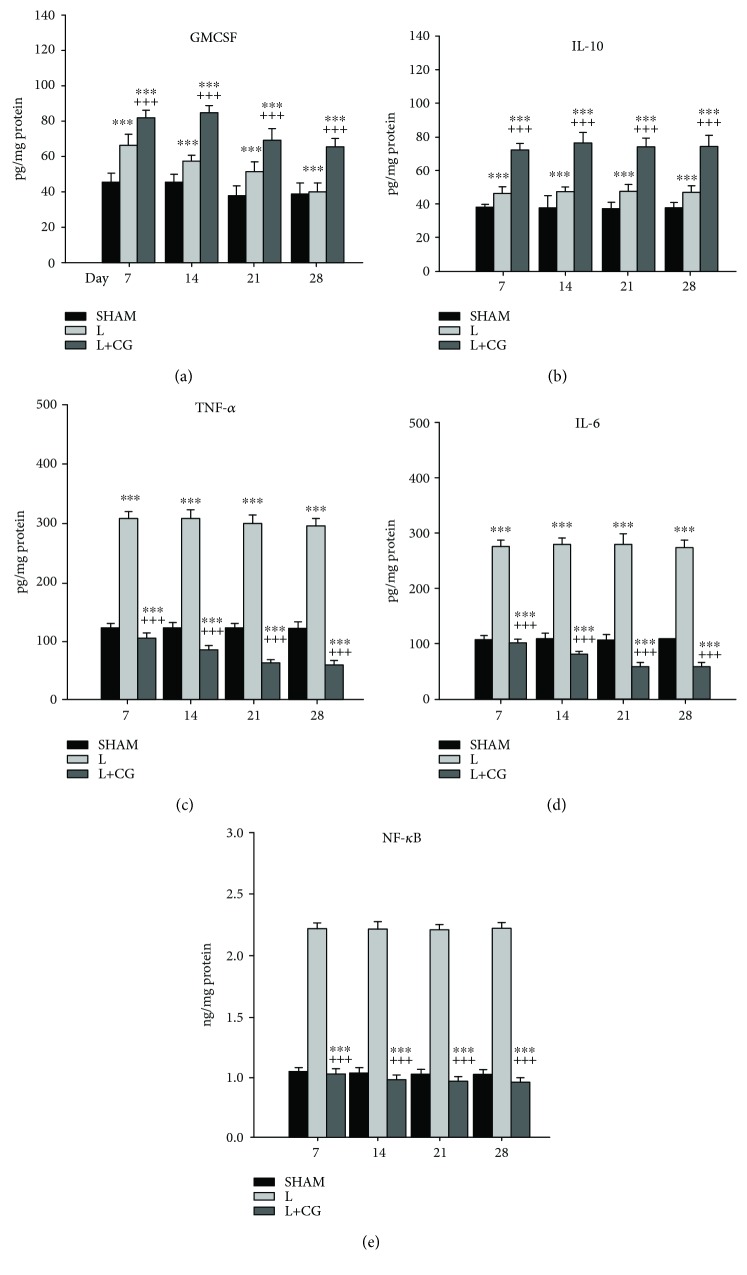
Changes in cytokine concentrations within brain tissue in the treatment groups. (a) Granulocyte-macrophage colony-stimulating factor (GMCSF), (b) interleukin-10 (IL-10), (c) tumor necrosis factor-alpha (TNF-*α*), (d) IL-6, and (e) NF-*κ*B (nuclear factor kappa-light-chain-enhancer of activated B cells) protein concentration as measured by ELISA in the brain tissue from the sham (SHAM), surgery-induced brain lesion (L), and collagen-glycosaminoglycan matrix implantation following surgery-induced lesion cavity (L+CG) groups. Data are presented as the mean ± standard deviation. ^∗^*p* < 0.05, ^∗∗^*p* < 0.01, and ^∗∗∗^*p* < 0.001 vs. the SHAM group; ^+^*p* < 0.05, ^++^*p* < 0.01, and ^+++^*p* < 0.001 vs. the L+CG group.

The tissue concentrations of GMCSF, IL-10, TNF-*α*, IL-6, and NF-*κ*B were measured in the LBZ of the SHAM, L, and L+CG groups on D7, D14, D21, and D28 after the surgical induction of a brain lesion. The L+CG group showed significant increases (1.5-2-fold) compared with the SHAM and L groups, in the tissue concentration of TNF-*α*, IL-6, and NF-*κ*B on D7 (Figures 5(c)–5(e)). The levels of TNF-*α*, IL-6, and NF-*κ*B peaked on D21 in the L+CG group, and they were increased 3-4-fold compared with the SHAM and L groups. These increases were also observed on D28. The levels of GMCSF and IL-10 peaked on D7, and they were significantly increased in the L+CG group compared with the SHAM and the L groups; this increase was maintained until D28 (Figures 5(a) and 5(b)).

## 4. Discussion

The neural extracellular matrix (ECM) is mainly composed of hyaluronans, glycoproteins, and proteoglycans. Proteoglycans contribute to a large portion of the ECM in the central nervous system (CNS). These molecules contain a core protein which bonds covalently to repeating disaccharide units called glycosaminoglycans (GAG). Proteoglycans include membrane-associated heparin sulfate proteoglycans and chondroitin sulfate proteoglycans which are found in the pericellular space. Chondroitin sulfate proteoglycans are considered the main inhibitory component of the glial scar and thereby an attractive target for CNS repair. The previous reports have proposed that the collagen-glycosaminoglycan (CG) matrix serves as a moderator of cell behaviors, such as adhesion, growth, and differentiation, as well as offering structural support. Furthermore, our studies have shown that the implantation of CG matrices may lead to the promotion of angiogenesis, neurogenesis, and functional recovery after surgical brain injury [2–4].

The present study applied CG matrices to lesion sites in rats following cortical injury obtained via surgical brain trauma. This treatment method resulted in reduced neuroinflammation. The histological sections and chemokine changes demonstrated that the neuroprotective effect of the CG matrix in SBI may be due to its activity as a biological scaffold for the ECM. Implantation of the collagen matrix within the injured cavity appeared to decrease neural inflammation and injury. The collagen matrix was large enough to fit into the injured cavity following SBI and provided neuroprotective effects in the present study. Future studies are needed to elucidate the mechanisms by which the collagen matrix conferred the benefits observed in the present study.

The majority of the previous studies involving the use of biological scaffold matrices in experimental brain injury models have used a method in which stem cells are implanted in the local environment, with the addition of exogenous trophic factors [11]. Impregnation of growth factors within the ECM or delivery of trophic factors by microspheres optimizes the microenvironment for neural growth and stimulates neurogenesis, proliferation, migration, and neural survival. Collagen matrices with the impregnated nerve growth factor and brain-derived neurotrophic factor (BDNF) can enhance neurite outgrowth [12]. In addition, microspheres that release the basic fibroblast growth factor can improve cell survival and behavioral recovery after cerebral ischemia [13]. The previous study by the authors reported that the implantation of a collagen matrix can increase BDNF and glial cell-derived neurotrophic factor [2], leading to increased cell proliferation and functional recovery.

The use of collagen as a scaffold without the simultaneous presence of trophic factors has been shown to facilitate the survival and differentiation of neuronal stem cells after local transplantation [2, 10, 14]. The collagen matrix by itself may result in neural growth and functional recovery. Cellular and denaturized collagen matrices have been shown to promote neural growth [2, 15, 16], which suggests that these matrices alone may be biologically active. An *in vitro* study of collagen matrices has demonstrated the ability of the synthetic collagen matrix to support the growth of axons and dendrites in neuronal cultures [16]. The number of migratory neural precursors and proliferative cells increased over time after collagen matrix implantation following SBI in rats [2]. Collagen matrices have also been reported to enhance recovery from injury states in rats via the implantation of collagen scaffolds after surgical trauma, without growth factors or stem cell seeding. Decreased lesion volume and improved neurological function were observed; these were assessed using the tactile adhesive removal test and modified neurological severity scores [2].

While many similarities exist between the different neuronal injury models, there is a clear difference between the mechanisms of injury in SBI compared with surgical trauma. Unlike surgical trauma where the tissue is severed, traumatic brain injury also induces tissue destruction via the more complex injury method of sudden compressive force. Traumatic brain injury causes injury to the tissue by direct impact (contusion), as well as white matter degeneration away from the site of impact [17]. These mechanisms of injury are different at the macroscopic and cellular level. Although prior studies reporting neuroregenerative benefits of collagen matrices in surgical injury have been promising, its use in SBI has not yet been explored.

In the past, the ability of biological scaffolds to promote tissue repair has been extensively studied. Biological scaffolds, such as porcine urinary bladder ECM, have chemoattractant and mitogenic properties [18, 19]. Studies using ECM in neural tissues found differences in the material properties as well as the neurotrophic and chemotactic properties of ECMs from different sources [20, 21]. ECMs applied to the CNS differentiate neural stem cells into neurons [22] and increase neurite length [21] compared with ECMs from the urinary bladder. In stroke injury, an ECM scaffold has been shown to enable the survival and integration of neural stem cells in the lesion cavity [23], as well as reducing the lesion volume and improving neurobehavioral recovery [15]. The previous studies suggested that the possible mechanism of functional recovery and reduced lesion was due to the migration and/or volume preservation of neural precursor cells. However, the present study provided another possible mechanism for the observed neuroprotective effects, specifically the suppression of microglial activation and inhibition of inflammatory cytokines.

Increased neuronal growth at the site of contusion injury may attenuate functional deficits in the hippocampal or cortical tissue and may lead to improvements in functional outcome, as well as reductions in lesion volume. The ability of collagen to stimulate neurogenesis and improve neural growth, differentiation, and survival is likely multifactorial. Collagen itself has chemotactic properties, as when it is actively degraded it attracts fibroblasts and promotes colonization [24]. As no collagen matrix was visualized in its original form at the time of dissection in the present study, it was likely degraded over time, with the byproducts of this degradation promoting recovery in the perilesional areas. Another possibility is the integration of the collagen matrix into the brain parenchyma. The high porosity of the graft is thought to aid fibroblast migration and vascularization. The collagen sponge dural graft has been shown to strengthen the integration of the graft with native tissue in the previous study [25]. Collagen has also been shown to enhance the survival of CNS neurons and suppress apoptosis in culture [26, 27]. However, it is unknown whether these are the mechanisms that resulted in the neuroprotective effects and reduction of neuroinflammation observed in the present study. The results of the current study show that cell survival is enhanced following implantation of the collagen matrix. Further investigation with various signaling pathways, including the downregulation of microglia activation and proinflammatory cytokines, is needed to verify the specific mechanisms of CG matrix neuroprotection.

The involvement of pro- and anti-inflammatory cytokines and chemokines in SBI is closely associated with neuroinflammation and neuroproduction. The neuroinflammatory cascade, which is activated in response to SBI, is mediated by the release of pro- and anti-inflammatory cytokines and chemokines. The microglia are the primary source of these inflammatory mediators within the brain. Gene profiling studies in experimental models of SBI have shown that genes related to neuroinflammation are strongly upregulated in the acute phase after SBI [28–30]. Additional studies have focused on microglial related gene localization after SBI. The results demonstrated that microglial marker activation (e.g., CD68 and MHCII), stress responses (e.g., p22phox and heme oxygenase 1), and chemokine expression (e.g., CXCL10 and CXCL6) were markedly increased after SBI [31]. These results are consistent with the early activation profile of microglia following injury. There is a rapid elevation of IL-1*β* and IL-6 proinflammatory cytokines within hours of SBI in humans and rodents [32–34]. Raised IL-6 levels correlate with improved outcomes after SBI [35]. The damaging effects of IL-1*β* are mediated via the IL-1 receptor type 1, which is expressed on microglia and neurons [36, 37]. Damage is not caused by the cytokine itself, but its activating effect on other proinflammatory signaling pathways, such as TNF-*α* [38]. Inhibiting IL-1*β* in experimental models of SBI has been shown to decrease inflammation and improve functional recovery [39–41]. IL-1*β* has potent neurotoxic effects and is also known to be synergistically enhanced by the presence of TNF-*α*, which suggests that these important cytokines mediate posttraumatic inflammation and brain damage [42]. IL-1*β* and TNF-*α* interact with distinct receptors that are structurally unrelated; however, both cytokines share significant downstream signaling pathways that may promote their synergistic action. TNF-*α* levels are consistently observed to be elevated in the serum and cerebrospinal fluid shortly after injury in severely injured SBI patients [43, 44]. The role of TNF-*α* in the pathogenesis of SBI is complex in nature with different functional outcomes in the acute and delayed phases after SBI [45, 46]. Initial preclinical neuroprotection studies targeting TNF-*α* showed considerable promise with three different compounds, including dexanabinol (HU-211), TNF-binding protein, and pentoxifylline, causing significant improvements in neurological outcomes and reducing posttraumatic BBB disruption and brain edema [47]. The results of the present study showed that NF-*κ*B, TNF-*α*, and IL-6 levels were elevated after SBI, which is consistent with the increased inflammatory cytokines observed in the previous studies. Furthermore, the CG matrix used in the present study can provide neuroprotective effects via the upregulation of IL-10 and GM-CSF and the downregulation of NF-*κ*B, TNF-*α*, and IL-6 (Figure 5).

There are several limitations of the present study design. First, we did not use different mixture doses to verify and confirm the real effect of our CG matrix. Second, we did not use any anti-inflammatory drugs to compare and confirm our matrix action in this basic research. Third, we did not perform reverse transcription polymerase chain reaction to evaluate mRNA levels through a real-time apparatus to understand the degree of inflammatory response together with the protein levels measured. Fourth, the implantation of the CG matrix occurred immediately following SBI, which may differ from a clinical scenario. In a setting of human SBI, the application of a neuroprotective scaffold may not occur for several hours after the initial injury when patients are brought into a clinical setting. However, the intention of the current experiment was to verify the neuroprotective benefits of the CG matrix after injury to the brain parenchyma. Fifth, a specific molecular mechanism underlying these improvements has not yet been elucidated. In the future studies, the mechanism of neuroprotection by the collagen matrix after SBI should be further investigated.

## 5. Conclusions

The results of the present study demonstrate that the implantation of CG scaffolds following surgical brain trauma has neuroprotective effects. Rats who received immediate implantation of the CG matrix at the site of SBI exhibited neuroprotective effects compared with the L and SHAM groups, as shown by the histological data.

## Data Availability

The datasets generated and analyzed during the current study are available from the corresponding author on reasonable request.

## References

[1] Zhang D., Yan H., Li H. (2015). TGF*β*-activated kinase 1 (TAK1) inhibition by 5Z-7-oxozeaenol attenuates early brain injury after experimental subarachnoid hemorrhage. *The Journal of Biological Chemistry*.

[2] Huang K. F., Hsu W. C., Chiu W. T., Wang J. Y. (2012). Functional improvement and neurogenesis after collagen-GAG matrix implantation into surgical brain trauma. *Biomaterials*.

[3] Hsu W. C., Yu C. H., Kung W. M., Huang K. F. (2018). Enhancement of matrix metalloproteinases 2 and 9 accompanied with neurogenesis following collagen glycosaminoglycan matrix implantation after surgical brain injury. *Neural Regeneration Research*.

[4] Huang K. F., Hsu W. C., Hsiao J. K., Chen G. S., Wang J. Y. (2014). Collagen-glycosaminoglycan matrix implantation promotes angiogenesis following surgical brain trauma. *BioMed Research International*.

[5] Harley B. A. C., Kim H.-D., Zaman M. H., Yannas I. V., Lauffenburger D. A., Gibson L. J. (2008). Microarchitecture of three-dimensional scaffolds influences cell migration behavior via junction interactions. *Biophysical Journal*.

[6] Hsu W. C., Spilker M. H., Yannas I. V., Rubin P. A. (2000). Inhibition of conjunctival scarring and contraction by a porous collagen-glycosaminoglycan implant. *Investigative Ophthalmology & Visual Science*.

[7] Yannas I. V., Lee E., Orgill D. P., Skrabut E. M., Murphy G. F. (1989). Synthesis and characterization of a model extracellular matrix that induces partial regeneration of adult mammalian skin. *Proceedings of the National Academy of Sciences of the United States of America*.

[8] Wu W.-C., Lai C. C., Chen H. S. L. (2008). Efficacy and safety of biodegradable collagen-glycosaminoglycan polymer as a material for scleral buckling. *Investigative Ophthalmology & Visual Science*.

[9] Kernie S. G., Parent J. M. (2010). Forebrain neurogenesis after focal ischemic and traumatic brain injury. *Neurobiology of Disease*.

[10] Li X., Zhao Y., Cheng S. (2017). Cetuximab modified collagen scaffold directs neurogenesis of injury-activated endogenous neural stem cells for acute spinal cord injury repair. *Biomaterials*.

[11] Zhao T., Yan W., Xu K., Qi Y., Dai X., Shi Z. (2013). Combined treatment with platelet-rich plasma and brain-derived neurotrophic factor-overexpressing bone marrow stromal cells supports axonal remyelination in a rat spinal cord hemi-section model. *Cytotherapy*.

[12] Labour M. N., Banc A., Tourrette A. (2012). Thick collagen-based 3D matrices including growth factors to induce neurite outgrowth. *Acta Biomaterialia*.

[13] Sun D., Wang W., Wang X. (2018). bFGF plays a neuroprotective role by suppressing excessive autophagy and apoptosis after transient global cerebral ischemia in rats. *Cell Death & Disease*.

[14] Fan C., Li X., Xiao Z. (2017). A modified collagen scaffold facilitates endogenous neurogenesis for acute spinal cord injury repair. *Acta Biomaterialia*.

[15] Chiono V., Tonda-Turo C. (2015). Trends in the design of nerve guidance channels in peripheral nerve tissue engineering. *Progress in Neurobiology*.

[16] Li X., Dai J. (2018). Bridging the gap with functional collagen scaffolds: tuning endogenous neural stem cells for severe spinal cord injury repair. *Biomaterials Science*.

[17] Wang H., Zhang Y. P., Cai J. (2016). A compact blast-induced traumatic brain injury model in mice. *Journal of Neuropathology & Experimental Neurology*.

[18] Wang Y., Papagerakis S., Faulk D. (2018). Extracellular matrix membrane induces cementoblastic/osteogenic properties of human periodontal ligament stem cells. *Frontiers in Physiology*.

[19] Huleihel L., Hussey G. S., Naranjo J. D. (2016). Matrix-bound nanovesicles within ECM bioscaffolds. *Science Advances*.

[20] Crapo P. M., Medberry C. J., Reing J. E. (2012). Biologic scaffolds composed of central nervous system extracellular matrix. *Biomaterials*.

[21] Medberry C. J., Crapo P. M., Siu B. F. (2013). Hydrogels derived from central nervous system extracellular matrix. *Biomaterials*.

[22] Crapo P. M., Tottey S., Slivka P. F., Badylak S. F. (2014). Effects of biologic scaffolds on human stem cells and implications for CNS tissue engineering. *Tissue Engineering Part A*.

[23] Bible E., Qutachi O., Chau D. Y. S., Alexander M. R., Shakesheff K. M., Modo M. (2012). Neo-vascularization of the stroke cavity by implantation of human neural stem cells on VEGF-releasing PLGA microparticles. *Biomaterials*.

[24] Kew S. J., Gwynne J. H., Enea D. (2012). Synthetic collagen fascicles for the regeneration of tendon tissue. *Acta Biomaterialia*.

[25] Lewis K. M., Sweet J., Wilson S. T., Rousselle S., Gulle H., Baumgartner B. (2018). Safety and efficacy of a novel, self-adhering dural substitute in a canine supratentorial durotomy model. *Neurosurgery*.

[26] Vecino E., Heller J. P., Veiga-Crespo P., Martin K. R., Fawcett J. W. (2015). Influence of extracellular matrix components on the expression of integrins and regeneration of adult retinal ganglion cells. *PLoS One*.

[27] Hopkins A. M., DeSimone E., Chwalek K., Kaplan D. L. (2015). 3D *in vitro* modeling of the central nervous system. *Progress in Neurobiology*.

[28] Shojo H., Borlongan C. V., Mabuchi T. (2017). Genetic and histological alterations reveal key role of prostaglandin synthase and cyclooxygenase 1 and 2 in traumatic brain injury-induced neuroinflammation in the cerebral cortex of rats exposed to moderate fluid percussion injury. *Cell Transplantation*.

[29] Rao J. S., Kellom M., Kim H. W., Rapoport S. I., Reese E. A. (2012). Neuroinflammation and synaptic loss. *Neurochemical Research*.

[30] Kumar A., Loane D. J. (2012). Neuroinflammation after traumatic brain injury: opportunities for therapeutic intervention. *Brain, Behavior, and Immunity*.

[31] Gyoneva S., Ransohoff R. M. (2015). Inflammatory reaction after traumatic brain injury: therapeutic potential of targeting cell-cell communication by chemokines. *Trends in Pharmacological Sciences*.

[32] Ray A. K., DuBois J. C., Gruber R. C. (2017). Loss of Gas6 and Axl signaling results in extensive axonal damage, motor deficits, prolonged neuroinflammation, and less remyelination following cuprizone exposure. *Glia*.

[33] Woodcock T., Morganti-Kossmann M. C. (2013). The role of markers of inflammation in traumatic brain injury. *Frontiers in Neurology*.

[34] Thelin E. P., Hall C. E., Gupta K. (2018). Elucidating pro-inflammatory cytokine responses after traumatic brain injury in a human stem cell model. *Journal of Neurotrauma*.

[35] Davidson J., Cusimano M. D., Bendena W. G. (2015). Post-traumatic brain injury: genetic susceptibility to outcome. *The Neuroscientist*.

[36] Murray K. N., Parry-Jones A. R., Allan S. M. (2015). Interleukin-1 and acute brain injury. *Frontiers in Cellular Neuroscience*.

[37] Greenhalgh A. D., Brough D., Robinson E. M., Girard S., Rothwell N. J., Allan S. M. (2012). Interleukin-1 receptor antagonist is beneficial after subarachnoid haemorrhage in rat by blocking haem-driven inflammatory pathology. *Disease Models & Mechanisms*.

[38] Ramesh G., MacLean A. G., Philipp M. T. (2013). Cytokines and chemokines at the crossroads of neuroinflammation, neurodegeneration, and neuropathic pain. *Mediators of Inflammation*.

[39] Swaroop S., Sengupta N., Suryawanshi A. R., Adlakha Y. K., Basu A. (2016). HSP60 plays a regulatory role in IL-1*β*-induced microglial inflammation via TLR4-p38 MAPK axis. *Journal of Neuroinflammation*.

[40] Xiao Y., Li G., Chen Y. (2018). Milk fat globule-epidermal growth factor-8 pretreatment attenuates apoptosis and inflammation via the integrin-*β*3 pathway after surgical brain injury in rats. *Frontiers in Neurology*.

[41] Liu F., Chen Y., Hu Q. (2015). MFGE8/integrin *β*3 pathway alleviates apoptosis and inflammation in early brain injury after subarachnoid hemorrhage in rats. *Experimental Neurology*.

[42] Olmos G., Lladó J. (2014). Tumor necrosis factor alpha: a link between neuroinflammation and excitotoxicity. *Mediators of Inflammation*.

[43] Jassam Y. N., Izzy S., Whalen M., McGavern D. B., el Khoury J. (2017). Neuroimmunology of traumatic brain injury: time for a paradigm shift. *Neuron*.

[44] Zeiler F. A., Thelin E. P., Czosnyka M., Hutchinson P. J., Menon D. K., Helmy A. (2017). Cerebrospinal fluid and microdialysis cytokines in severe traumatic brain injury: a scoping systematic review. *Frontiers in Neurology*.

[45] Baratz R., Tweedie D., Wang J. Y. (2015). Transiently lowering tumor necrosis factor-*α* synthesis ameliorates neuronal cell loss and cognitive impairments induced by minimal traumatic brain injury in mice. *Journal of Neuroinflammation*.

[46] Wang J. Y., Huang Y. N., Chiu C. C. (2016). Pomalidomide mitigates neuronal loss, neuroinflammation, and behavioral impairments induced by traumatic brain injury in rat. *Journal of Neuroinflammation*.

[47] Patterson Z. R., Holahan M. R. (2012). Understanding the neuroinflammatory response following concussion to develop treatment strategies. *Frontiers in Cellular Neuroscience*.

